# Post-COVID-19 cognitive symptoms in patients assisted by a teleassistance service: a retrospective cohort study

**DOI:** 10.3389/fpubh.2024.1282067

**Published:** 2024-04-16

**Authors:** Lívia Paula Freire Bonfim, Thais Rotsen Correa, Bruno Cabaleiro Cortizo Freire, Thais Marques Pedroso, Daniella Nunes Pereira, Thalita Baptisteli Fernandes, Luciane Kopittke, Clara Rodrigues Alves de Oliveira, Antonio Lucio Teixeira, Milena Soriano Marcolino

**Affiliations:** ^1^Tropical Medicine and Infectious Disease Program, Faculdade de Medicina, Universidade Federal de Minas Gerais, Belo Horizonte, Minas Gerais, Brazil; ^2^Statistics Department, Institute of Exact Sciences, Universidade Federal de Minas Gerais, Belo Horizonte, Minas Gerais, Brazil; ^3^Telehealth Center, University Hospital, Universidade Federal de Minas Gerais, Belo Horizonte, Minas Gerais, Brazil; ^4^Faculdade de Ciências Médicas de Minas Gerais (FCMMG), Belo Horizonte, Minas Gerais, Brazil; ^5^Hospital Nossa Senhora da Conceição, Porto Alegre, Rio Grande do Sul, Brazil; ^6^Neuropsychiatry Program, Department of Psychiatry and Behavioral Sciences, UT Health Houston, Houston, TX, United States; ^7^National Institute for Health Technology Assessment (IATS), Porto Alegre, Rio Grande do Sul, Brazil

**Keywords:** post-acute COVID-19 syndrome, mental fatigue, cognitive dysfunction, memory disorders, risk factors

## Abstract

**Introduction:**

Four years after the onset of the COVID-19 pandemic, the frequency of long-term post-COVID-19 cognitive symptoms is a matter of concern given the impact it may have on the work and quality of life of affected people.

**Objective:**

To evaluate the incidence of post-acute COVID-19 cognitive symptoms, as well as the associated risk factors.

**Methods:**

Retrospective cohort, including outpatients with laboratory-confirmed COVID-19 and who were assisted by a public telehealth service provided by the Telehealth Network of Minas Gerais (TNMG), during the acute phase of the disease, between December/2020 and March/2022. Data were collected through a structured questionnaire, applied via phone calls, regarding the persistence of COVID-19 symptoms after 12 weeks of the disease. Cognitive symptoms were defined as any of the following: memory loss, problems concentrating, word finding difficulties, and difficulty thinking clearly.

**Results:**

From 630 patients who responded to the questionnaire, 23.7% presented cognitive symptoms at 12 weeks after infection. These patients had a higher median age (33 [IQR 25–46] vs. 30 [IQR 24–42] years-old, *p* = 0.042) with a higher prevalence in the female sex (80.5% vs. 62.2%, *p* < 0.001) when compared to those who did not present cognitive symptoms, as well as a lower prevalence of smoking (8.7% vs. 16.2%, *p* = 0.024). Furthermore, patients with persistent cognitive symptoms were more likely to have been infected during the second wave of COVID-19 rather than the third (31.0% vs. 21.3%, *p* = 0.014). Patients who needed to seek in-person care during the acute phase of the disease were more likely to report post-acute cognitive symptoms (21.5% vs. 9.3%, *p* < 0,001). In multivariate logistic regression analysis, cognitive symptoms were associated with female sex (OR 2.24, CI 95% 1.41–3.57), fatigue (OR 2.33, CI 95% 1.19–4.56), depression (OR 5.37, CI 95% 2.19–13.15) and the need for seek in-person care during acute COVID-19 (OR 2.23, CI 95% 1.30–3.81).

**Conclusion:**

In this retrospective cohort of patients with mostly mild COVID-19, cognitive symptoms were present in 23.7% of patients with COVID-19 at 12 weeks after infection. Female sex, fatigue, depression and the need to seek in-person care during acute COVID-19 were the risk factors independently associated with this condition.

## Introduction

With the emergence of the coronavirus disease 2019 (COVID-19), the world has been forced to face a health crisis that has lasted for over 4 years. Although current case numbers have drastically reduced, COVID-19 is still a global health issue, with a high social burden, especially in developing countries (1). In this context, the COVID-19 pandemic represented a great stimulus for the development of telehealth services worldwide, as the pandemic necessitated innovative solutions to provide quality health care while preserving protective social distancing measures – in place at the time – for the safety and of both health care providers and the general population.

Currently, increasing attention is directed towards the burden associated with symptoms that persist beyond the acute phase of the infection (2). According to the National Institute for Health and Care Excellence (NICE), post-COVID-19 syndrome refers to signs and symptoms that develop during or after an infection consistent with COVID-19, continue for at least 12 weeks and are not explained by an alternative diagnosis (3). Post-COVID-19 syndrome may occur regardless of acute COVID-19 severity, although those who present severe COVID-19 or have several comorbidities are more prone to develop the syndrome (1, 4).

The symptoms of post-COVID-19 syndrome are heterogeneous, and may include, but are not limited to, fatigue, difficulty breathing, “brain fog,” insomnia, joint pain, and cardiac issues (5). “Brain fog” is an umbrella term for the presence of cognitive symptoms, including mental fatigue, impaired concentration and memory that may impact daily activities. Fatigue and cognitive impairment, including “brain fog,” are among the most common and debilitating long-term effects of COVID-19 (6). An Israeli cohort examined the long-term clinical outcomes in over 1.9 million people with mild COVID-19, and observed that unvaccinated SARS-CoV-2 infected patients had a higher risk for concentration and memory impairment hazard ratio 1.85, 95% confidence interval [CI] 1.58 to 2.17 – between 1 and 6 months after acute infection; and hazard ratio 12.8, 95% confidence interval 9.6 to 16.1 – 6 months to a year after COVID-19 diagnosis, when compared to uninfected people (7). Other neurological and neuropsychiatric symptoms such as anxiety and depression are also common components of the post-COVID-19 syndrome (8).

The increase in the incidence of cognitive symptoms is a matter of concern given the impact they may have on the work and quality of life of the affected patients. However, little is known about its determinants, especially in patients who have had mild cases of SARS-CoV-2 infection (9). Therefore, this study aimed to evaluate the incidence of cognitive symptoms in post-COVID syndrome, as well as the associated risk factors, in COVID-19 outpatients assisted by a Brazilian public telehealth service. The study is innovative for including patients attended via a telehealth service during acute COVID-19.

## Methods

### Study design and eligibility

This is a retrospective cohort, which included staff and students from a public university, the *Universidade Federal de Minas Gerais* (UFMG), located in Belo Horizonte, the capital of Minas Gerais state, in Southeast Brazil. The cohort consisted of a convenience sample of consecutive individuals who had laboratory-confirmed COVID-19 (by real-time reverse transcription-polymerase chain reaction assay [RT-PCR] or antigen testing), between December 01, 2020 and March 31, 2022. All patients had been followed during the acute phase of the disease by TeleCOVID-MG, a public telehealth service provided by the Telehealth Network of Minas Gerais (TNMG) (10). The study period comprehended patients who had acute COVID-19 during the second or third pandemic waves in Brazil. According to previous evidence, the second wave lasted from August 11, 2020, to December 25, 2021, with delta and gamma variants as dominant; and the third wave lasted from December 26, 2021, to May 5, 2022, marked predominantly by the omicron variant.

### TeleCOVID-MG

TeleCOVID-MG was a public structured multilevel teleconsultation and telemonitoring program, developed by the TNMG, to assist patients with respiratory tract symptoms during the COVID-19 pandemic. This service was maintained in operation between May 2020 and March 2023. The TNMG represents a partnership between seven public Brazilian Universities, with a coordinating hub at the Telehealth Center at the University Hospital/UFMG (10). It is one of the largest telehealth services in Brazil and Latin America. TeleCOVID-MG was first implemented in two Brazilian medium-sized cities and then, in December 2020, it was expanded to assist students, faculty, and technical-administrative staff from UFMG, as well as healthcare professionals from UFMG’s University Hospital. *Universidade Federal de Minas Gerais* is a public federal university with more than 40,000 students (undergraduate and postgraduate), and the staff has more than 10,000 people, including professors and administrative staff.

For the UFMG students and staff, access to the TeleCOVID-MG service was done through an online symptom auto-verification application developed by the pandemic committee of the university. Upon identification of any flu-like symptoms, patients were referred to a chatbot, a computer program that collected name, Brazilian identification number, telephone number, warning signs or any comorbidities which increased the risk of worse outcomes (11). In cases of suspicion of flu-like syndrome and according to the severity of the symptoms, the patient was assisted by a nurse or a physician through a phone call teleconsultation. At the end of the teleconsultation, the patient was advised to keep domiciliary isolation or to seek an onsite evaluation at the primary care center or at the emergency department, in case of warning signs such as fever for more than 3 days, signs of hemodynamic instability, decompensation of the underlying disease or any other critical clinical condition identified by the health professional. In addition, patients received a request for an RT-PCR laboratory test to identify SARS-CoV-2, which could be performed at the university itself or at the reference laboratory of the patient’s preference. Positive antigen tests performed in duly accredited services were also accepted as laboratory confirmation for COVID-19 (12).

### Data collection

For the present study, data was collected through two main steps. In the first step, we obtained information on the COVID-19 acute phase, while in the second we assessed post-acute COVID-19 symptoms. Data regarding the acute phase of COVID-19 was obtained from the TeleCOVID-MG database including: age, sex, COVID-19 acute symptoms, the date on which the laboratory exam was performed, comorbidities, and if the patient was vaccinated for COVID-19 before the laboratory confirmation. With regards to the post-acute disease stage, data was collected at least 6 months after the diagnostic laboratory test, through the application of a structured questionnaire, developed exclusively for this study. The questionnaire was based on clinical protocols for the management of post-COVID syndrome, by the Brazilian Ministry of Health and the health department of Belo Horizonte (13, 14), as well as on previously validated tools, including Generalized Anxiety Disorder–7 (GAD-7) (15), Posttraumatic Stress Disorder Checklist for DSM-5 (PCL-5) (16), Patient Health Questionnaire-9 (PHQ-9) (17), Chalder’s Fatigue Scale (18, 19), New York Health Association’s functional scale (20), and Charlson’s comorbidity index (21).

The questionnaire was developed using *Google Forms*^®^ and was composed of 82 questions, divided into twelve sections: researcher identification, demographic characteristics, respiratory manifestations, neuromusculoskeletal disorders, physical fatigue, mental fatigue, neuro-cognitive manifestations, other manifestations, comorbidities, life habits (smoking and physical activity), work impact and post-COVID-19 functioning (Supplementary file 1). The questionnaire investigated symptoms at different time points after the COVID-19 acute phase (up to 1 month, up to 3 months, up to 6 months, more than 6 months). For the present analysis, the occurrence of cognitive symptoms for at least 12 weeks was evaluated.

The constructs used to assess cognitive functions, namely memory problems, concentration problems, difficulties for thinking clearly and word finding difficulties, were obtained from the Chalder Fatigue Scale (18, 19). This is a previously validated instrument widely applied to measure physical and mental fatigue in patients with chronic fatigue syndrome (18, 19). Emerging literature has linked post-COVID cognitive symptoms to myalgic encephalomyelitis/chronic fatigue syndrome (ME/CFS) in which the Chalder Fatigue Scale is frequently used (22). The question “Did you have difficulties in thinking clearly?” was adapted from the Chalder Fatigue Scale’s question: “Do you think as clearly as usual?.” Similarly, the question “Did you have word finding difficulties?” was adapted from the Chalder Fatigue Scale: “Do you find it more difficult to find the correct word?.” The question “Did you present memory loss?” was adapted from “Is your memory as good as usual?” and “Did you present alterations in concentration?” from “Do you have difficulty concentrating?” [(18), Supplementary file 1].

In the present study, the presence of cognitive symptoms in post-COVID-19 syndrome was considered if the patient had a positive response to at least one of the four questions related to the occurrence of memory problems, concentration problems, difficulties for thinking clearly and word finding difficulties. Other cognitive studies on post COVID have used a similar approach (23–25).

Regarding the presence of cognitive symptoms, patients were divided into two groups: those who presented at least one of the four cognitive symptoms (memory problems, concentration problems, word finding difficulties and difficulty in thinking clearly) and those who had no cognitive symptoms lasting at least 12 weeks from the onset of COVID-19 symptoms.

To evaluate neuropsychiatric manifestations, questions based on previously validated instruments (GAD-7 for anxiety, PCL-5 for post-traumatic disorder and PHQ-9 for depression) were included in the questionnaire (15–17).The PHQ-2, which is an abbreviated form of the PHQ-9, was used as a criterion for the occurrence of depression. In other words, the positive answer to the two PHQ-2 questions defined the occurrence of depression in the present study (questions 49 and 54 of Supplementary file 1).

Patients who practiced physical activities regularly in accordance with World Health Organization recommendations (at least 150 min of physical exercise at moderate intensity or 75 at vigorous intensity, weekly) were considered non-sedentary (26).

In order to assess the possible impact caused by the post-COVID-19 syndrome, patients were asked about eventual loss of ability to carry out their daily tasks, the need to leave work longer than the expected period of isolation (for the acute phase of COVID-19), as well as the need for any restrictions after returning to work, such as reducing the workload or adapting the activity carried out. Finally, survey participants rated themselves on a post-COVID functional status scale (13, 27).

The questionnaire was applied through phone calls by a team of eight trained researchers who were supervised by a senior researcher. A data collection protocol (Supplementary file 2) was created in order to standardize the collection and all team members were previously trained on the study protocol.

The questionnaire was applied from December 2021 to November 2022. Each participant received a single phone call at least 6 months after laboratory confirmation of COVID-19. The protocol for patient inclusion in the study involved four contact attempts, including two phone calls, one in the morning and one in the afternoon, and two standardized text messages through an app (*Whatsapp*^®^). This message consisted of a short text presenting the project, and the individual was inquired about the best time for the telephone call. If the participant initially did not understand any question, as per the study protocol the researcher should explain the question, according to the collection manual. As the questionnaires were filled out, the senior researcher audited the responses. Periodic audits were performed weekly, in order to increase quality data and to reduce biases. Incorrect data were reported to applicators and corrected. Whenever necessary, researchers underwent refresher training before applying new questionnaires.

**Figure 1:**
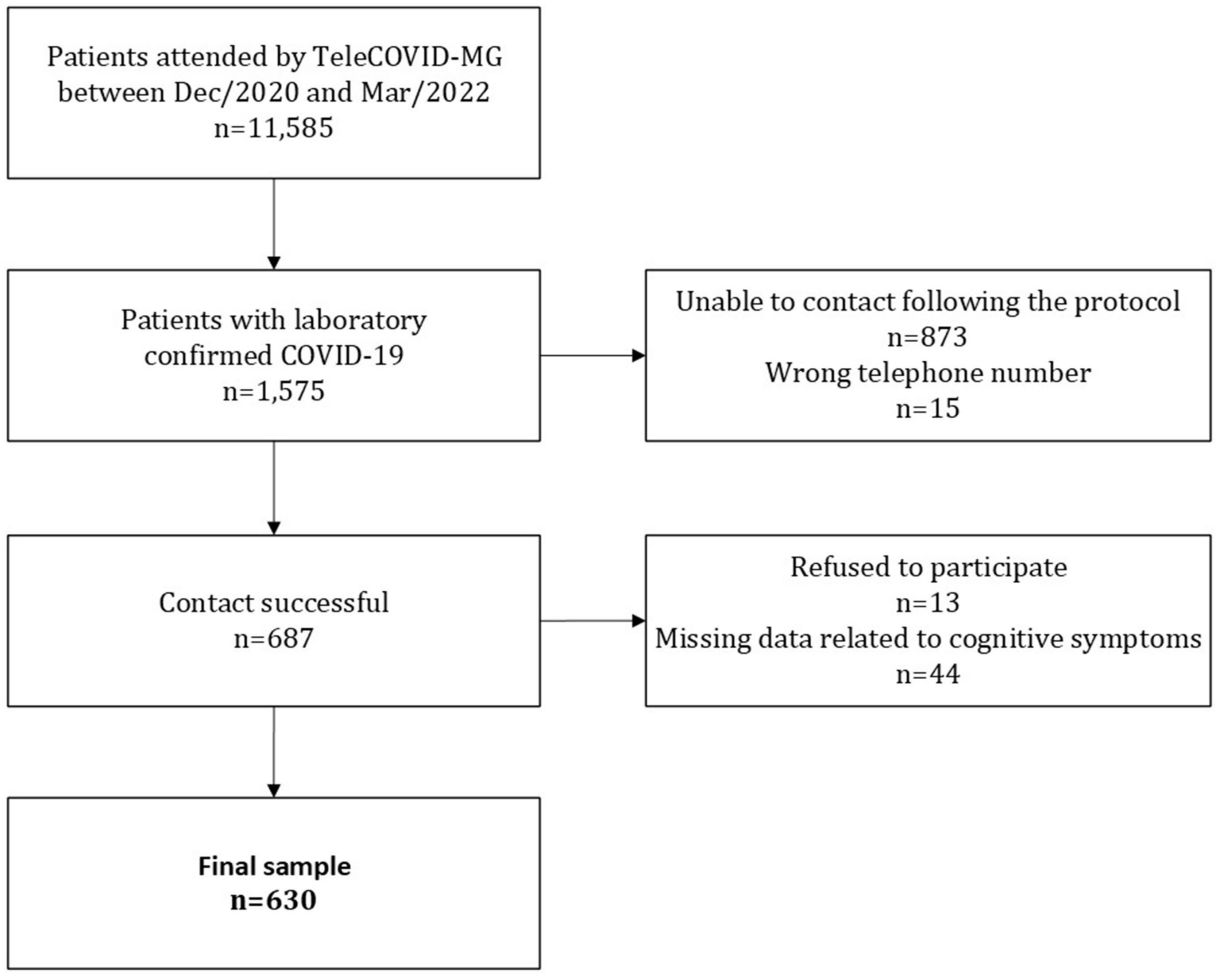
Flowchart of patients included in the study.

During the study period, 11,585 patients were treated by TeleCOVID-MG. Of these, 1,575 had a positive laboratory test (RT-PCR or antigen test) for SARS-CoV-2. All patients with positive tests in this period would be able to participate in the research through the application of the questionnaire on post-COVID symptoms. However, in 888 cases contact was unsuccessful, of these 873 patients did not answer calls, and 15 phone numbers were wrong. Among 687 patients who answered the call, 13 refused to participate in the study, and 44 were excluded by missing data in all questions concerning cognitive symptoms. In the end, a total of 630 participants were included in this study (Figure 1).

### Statistical analysis

Descriptive analysis of the variables regarding the presence or absence of cognitive symptoms, patient characteristics and impact on daily activities was performed. Participants were categorized into three age groups: 17–40, 41–60, and >60 years (7). The wave the patient was infected was defined based on the date of the laboratory examination. Exams taken between November 8, 2020 and December 25, 2021 corresponded to the second wave; while the tests carried out between December 26, 2021 and March 31, 2022 referred to the third wave of COVID-19 in Brazil (28).

For the purpose of the current analysis, the number of comorbidities was defined according to a modified Charlson comorbidity index including: chronic cardiac disease, chronic respiratory disease (excluding asthma), chronic renal disease, liver disease, dementia, chronic neurological conditions, connective tissue disease, diabetes mellitus, HIV and malignancy (21). Obesity was not included in the original modified Charlson comorbidity index, but we opted to include it due to its probable association with adverse outcomes in patients with COVID-19.

The statistical method to analyze the association between patient characteristics and the occurrence of cognitive symptoms (each symptom separately and also at least one of the four symptoms) was based on Bursac et al.’s proposal (29). The authors suggest starting the selection of variables through an univate analysis with a cutoff *p*-value of 0.25, but we opted to be more strict and 0.20 as the cutoff point.

The selected characteristics (possible predictors of cognitive symptoms) were then included in multivariate logistic regression models. Variables that were considered possible consequences of cognitive symptoms or other post-COVID-19 manifestations (such as loss of ability to perform daily tasks, absence from work longer than the usual period of isolation, restrictions on return to work and functional status post-COVID) were not tested in the multivariate models (Supplementary Table S1). A backward stepwise method was used to define significant characteristics, considering a cutoff point of 5%. The impact of significant characteristics was estimated using odds ratios (OR) and 95% confidence intervals. Deviance, Pearson, Hosmer-Lemeshow indicated well-adjusted final models.

As the presence of depression is linked to cognitive impairments, and a higher frequency of cognitive symptoms not always confirmed by the objective assessment (30), a subanalysis was conducted, excluding patients with depression.

### Ethics statement

This study was approved by the Brazilian National Commission for Research Ethics (*Comissão Nacional de Ética em Pesquisa* on number CAAE 30350820.5.1001.0008), and it was conducted in accordance with the Declaration of Helsinki. All patients gave informed consent to participate in the study.

## Results

### Study population

Of the 630 post-COVID-19 patients included in the study, 149 (23.7%) reported cognitive symptoms at least 12 weeks after COVID-19 infection. The main characteristics of the study’s population stratified by the presence of cognitive symptoms are presented in Table 1 and Supplementary Table S2.

**Table 1:** Baseline characteristics of the study cohort at 12 weeks after acute COVID-19 onset.

Characteristics	Total sample (*n* = 630)		Cognitive symptoms (*n* = 149)		No cognitive symptoms (*n* = 481)		*p*-value
Age (years)	31	(24–43)	33	(25–46)	30	(24-42)	**0.042**
Women	419	(66.5)	120	(80.5)	299	(62.2)	**<0.001**
Pregnancy	6	(1.4)	2	(1.7)	4	(1.3)	0.798
Healthcare Professional	127	(20.2)	37	(24.8)	90	(18.7)	0.202
Vaccination status							0.518
Two vaccine doses	416	(66.0)	102	(68.5)	314	(65.3)	
Partially vaccinated	121	(19.2)	25	(16.8)	96	(20.0)	
Unvaccinated	57	(9.0)	16	(10.7)	41	(8.5)	
Missing data	36	(5.7)	NA		NA		
COVID-19 wave							**0.014**
Second wave	155	(24.6)	48	(32.2)	107	(22.2)	
Third wave	474	(75.2)	101	(67.8)	373	(77.5)	
Other	1	(0.2)	0		1	(0.2)	
Modified Charlson Index							0.491
0 comorbidity	540	(85.7)	130	(87.2)	410	(85.2)	
1 comorbidity	85	(13.5)	17	(11.4)	68	(14.1)	
2 comorbidities	5	(0.8)	2	(1.3)	3	(0.6)	
Depression	25	(4.0)	17	(11.4)	8	(1.7)	**<0.001**
Fatigue	524	(83.2)	138	(92.6)	386	(80.2)	**<0.001**
Smoking	90	(14.3)	13	(8.7)	77	(16.0)	**0.026**
Sedentary lifestyle	266	(42.2)	72	(48.3)	194	(40.3)	0.084
Needed to seek in-person care	76	(12.1)	32	(21.5)	44	(9.1)	**<0.001**
Hospital admission	3	(0.5)	2	(1.3)	1	(0.2)	**1.000**
Started treatment for psychiatric diseases	82	(13.0)	32	(21.5)	50	(10.4)	**<0.001**
Loss of ability to carry out daily tasks	31	(4.9)	25	(16.8)	6	(1.2)	**<0.001**
Time away from work longer than the usual period of isolation	19	(3.0)	11	(7.4)	8	(1.7)	**<0.001**
Restrictions on returning to work	8	(1.3)	6	(4.0)	2	(0.4)	**<0.001**
Post-COVID functional status^a^							**<0.001**
No impairment	479	(76.0)	53	(35.6)	426	(88.6)	
Very mild impairment	112	(17.8)	66	(44.3)	46	(9.6)	
Mild impairment	38	(6.0)	30	(20.1)	8	(1.7)	
Moderate impairment	1	(0.2)	0		1	(0.2)	

Numbers: N (%) or median (IQR); IQR: interquartile range; NA: not applicable.

^a^No patients had severe impairment.

The median age and proportion of women were higher in the group of patients with cognitive symptoms when compared to those without cognitive symptoms (33 [interquartile range (IQR) 25–46] vs. 30 [IQR 24–42] years-old, *p* = 0.042; 80.5% vs. 62.2%, *p* < 0.001). With regards to race, education, being a healthcare worker or intern, vaccination status, pregnancy, and physical activity, there were no statistically significant differences between groups. As for the presence of comorbidities, the vast majority of the sample (85.7%) did not present any comorbidity, and, among those who reported at least one comorbidity, there was no difference between groups. Patients with cognitive symptoms had a higher frequency of symptoms related to depression (11.4% vs. 1.7, *p* < 0.001), lower frequency of smoking (8.7% vs. 16.0%, *p* = 0.026), higher frequency of infection during the second wave (32.2 vs. 22.2%, *p* = 0.014) and sought in-person care more frequently (21.5% vs. 9.1%, *p* < 0.001), when compared to those without cognitive symptoms. When assessing patients per COVID-19 wave (Supplementary Table S3), there was a higher frequency of cognitive symptoms in the second wave.

Patients with cognitive symptoms reported a higher frequency of starting treatment for psychiatric diseases (21.5% vs. 10.4%, *p* < 0.001), greater loss of ability to perform day-to-day tasks (16.8% vs. 1.2%, *p* < 0.001), needed to be absent from work activities for a longer period (7.4% vs. 1.7%, *p* < 0.001) and reported having more limitations than patients without cognitive symptoms (44.3% vs. 9.6% to no impairment, 20.1% vs. 1.7% to mild impairment, *p* < 0.001).

### Predictive factors for cognitive symptoms

The most prevalent cognitive symptoms in the sample studied were memory loss (17.5%), followed by word finding difficulties (16.2%), concentration problems (15.9%), and difficulty in thinking clearly (9.5%), as shown in Table 2. Among affected patients (*n* = 149), 23.5% had all four symptoms, 24.8% had three symptoms, 29.5% had two symptoms, and 22.1% had only one symptom.

**Table 2:** Incidence of cognitive symptoms in the study population.

Symptom	Total sample (*n* = 630)
Memory loss	110 (17.5)
Word finding difficulties	102 (16.2)
Concentration problems	100 (15.9)
Difficulty thinking clearly	60 (9.5)
At least one of the four	149 (23.7)

Numbers: *N* (%).

In the multivariate analysis, a statistically significant association was observed between the occurrence of cognitive symptoms in post-COVID-19 syndrome and depression (OR 5.37 [95% IC 2.19–13.15]), as well as the presence of fatigue (OR 2.33 [95% CI 1.19–4.56]), female sex (OR 2.24 [95% CI 1.41–3.57]) and the need to seek in-person care in the acute phase of COVID-19 infection (OR 2.23 [95% CI 1.30–3.81]) (Table 3). In regards to each symptom, the same factors were associated (depression, fatigue, sex and need to seek in-person care), except for “thinking clearly,” which showed no significant association with the need to seek in-person care, but had a significant association with COVID-19 acute infection during the second wave (OR 1.92 [95% CI 1.08–3.41]).

**Table 3:** Predictors of cognitive symptoms according to the multivariate analysis (*n* = 630).

Variable	At least one cognitive symptom	Concentration	Memory	Wordfindingdifficulties	Thinkclearly
Depression	5.37 (2.19–13.15)	6.72 (2.81–16.09)	3.76 (1.60–8.87)	2.81 (1.18–6.70)	4.31 (1.75–10.63)
Fatigue	2.33 (1.19–4.56)	3.23 (1.26–8.31)	3.87 (1.52–9.89)	2.35 (1.04–5.31)	3.93 (1.20–12.88)
Women	2.24 (1.41–3.57)	2.66 (1.48–4.78)	2.09 (1.24–3.54)	2.24 (1.29–3.87)	NA
Needed to seek in-person care	2.23 (1.30–3.81)	1.87 (1.02–3.43)	2.39 (1.36–4.20)	2.82 (1.61–4.95)	NA
COVID wave (second)	NA	NA	NA	NA	1.89 (1.07–3.34)

Numbers are presented as odds ratio (95% confidence interval); *p* < 0.05. NA: not applicable; *p* > 0.20 in the univariate analysis (Table 1).

In the subanalysis excluding patients with depressive symptoms (*n* = 25), results were similar to the previous model (Supplementary Table S4).

## Discussion

The present study found that cognitive symptoms are a prominent feature of post-COVID-19 syndrome, with a prevalence of 23.7%. Female sex OR 2.27 (95% CI [1.41–3.57]), fatigue OR 2.33 (95% CI [1.19–4.56]), depression OR 5.37 (95% CI [2.19–13.15]) and the need to seek in-person care in the acute phase of COVID-19 infection OR 2.23 (IC 95% [1.30–3.81]) were associated with cognitive symptoms. To the best of our knowledge, this is the first study in Latin America to address post-COVID-19 cognitive symptoms.

From the total sample, 12.1% required to seek in-person care and only 0.5% required hospitalization, which confirms that overall this is a cohort of mild cases of COVID-19. Even though the cases were mostly mild and patients were young, post-COVID-19 cognitive symptoms were reported by almost a quarter of them. This number corroborates previous studies in which cognitive symptoms, especially memory impairment, were highly prevalent among patients who had COVID-19 (6, 8, 31, 32). A recent systematic review, including data from 10,530 patients (59% women, average age 52 years, 51% who were hospitalized and 3% were admitted to an intensive care unit), has shown that cognitive symptoms were present in roughly one-third of patients at 12 or more weeks after the onset of COVID-19: brain fog (32, 10–54%), memory issues (28, 22–35%), attention disorder (22, 7–36%) (8). Interestingly, the prevalence of cognitive symptoms did not change significantly between mid-term (3 to 6 months) and long-term follow-up (6 or more months post-infection, lower than 5% change).

Women were 2.24 (95% CI 1.41–3.57) times more likely to have at least one of the four cognitive symptoms than men, with greater chances of having concentration problems (OR 2.66 [95% CI 1.48–4.78]), memory loss (2.09 [95% CI 1.24–3.54]), and word finding difficulties (2.24 [95% CI 1.29–3.87]). These findings are in line with two large cohorts in Iran and Norway, which also observed female sex as a risk factor for post-COVID brain fog (OR 1.4 [95% CI 1.06–1.90] and RR 2.0 [95% IC 1.3–3.2], respectively) (33, 34), and a recent Polish study with 303 outpatients, 47% of them healthcare professionals, and a median age similar to the present study. In this Polish study, 12 weeks after acute COVID-19, women reported problems with writing, reading, counting (17.0 vs. 5.1%) and communication of thoughts in a way that others can understand (34.3 vs. 20.7%) more often than men (35).

Patients who experienced fatigue were 2.33 (95% CI 1.19–4.56) times more likely to have cognitive symptoms than those who did not experience it, with 3.23 (95% CI [1.26–8.31]) greater chance of having concentration problems; 3.87 (95% CI 1.52–9.89) greater chance of reporting memory problems, 2.35 (95% CI 1.04–5.31) greater chance of reporting word finding difficulties and 3.40 (95% CI 1.03–11.25) greater chance of having difficulty thinking clearly. A systematic review and meta-analysis that included almost 50,000 patients from various countries and settings, with different levels of severity, observed that approximately one-third of subjects experienced persistent post-COVID-19 fatigue and more than one-fifth of subjects exhibited cognitive impairment 12 or more weeks after confirming the diagnosis of acute COVID-19 (8).

Regarding the severity of disease, patients who needed to seek in-person care were 2.23 (95% CI 1.30–3.81) times more likely to have cognitive symptoms than those who did not, with 2.87 (CI 95% 1.02–4.43) greater chance of having concentration problems; 2.39 (CI 95% 1.36–4.20) greater chance of reporting memory problems and 2.82 (95% CI 1.61–4.95) greater chance of reporting word finding difficulties. This result agrees with the trend that the more severe the acute infection, the greater the chance of developing cognitive symptoms as a feature of the post-COVID-19 syndrome (36). In an ongoing cohort study that followed more than 70,000 adult participants during the COVID-19 pandemic, there was a higher prevalence of cognitive symptoms among individuals with moderate/severe COVID-19 when compared to mild cases (RR 1.9 [95% CI 1.3–2.9]) (34). In addition, a recent North American study of 89 patients hospitalized during acute SARS-CoV-2 infection with 6 months of follow-up found that having developed pneumonia after COVID-19 is a risk factor for cognitive symptoms (OR 1.69 [95% CI 1.16–2.46]) (37).

As for medical comorbidities, the presence of one or more of them did not influence the occurrence of persistent cognitive symptoms. This could be due to the nature of the sample, which consisted of patients with mild COVID-19. A systematic review and meta-analysis including 677,045 COVID-19 survivors demonstrated that underlying comorbidities may be a predisposing factor for the development of long-term COVID-19 symptoms (38). However, studies specifically assessing comorbidities as a risk factor for post-COVID-19 cognitive symptoms are needed.

Although the pathophysiology underlying post-COVID-19 cognitive symptoms is not understood, there are interesting. In the acute phase of the disease, the SARS-CoV-2 virus can penetrate the blood–brain barrier directly through the olfactory nerve, and viral proliferation can benefit from areas of cerebral hypoxia, increasing the central nervous system (CNS) viral load over time and affecting mitochondrial function. As brain tissues have high metabolic demand, this can lead to cognitive impairment (39). Another theory suggests that the impairment is not caused by a direct viral aggression to the CNS but by an overreaction of the immune system’s response to the infection (40). It is also possible that those symptoms are the consequence of acute phase damage since the severity of acute disease is associated with cognitive symptoms (32). Finally, it is important to highlight that the inflammation and oxidative stress due to the SARS-CoV-2 infection may lead to neuropathological processes, such as cortical and hippocampal atrophy and small vessel disease, which could contribute to post-COVID-19 cognitive dysfunction symptoms (6). It should be noted that while symptoms related to concentration, language and memory have relatively well-defined neuroanatomical correlates, “thinking clearly” cannot be easily mapped into some brain neural circuitry or structure (41).

Cognitive subdomains such as memory and concentration are significantly impaired during and between episodes in individuals with depression (42). Multiple interacting neurobiological mechanisms (e.g., neuroinflammation and endothelial dysfunction) are implicated as subservient to cognitive deficits in depressive episodes (42, 43). In the current study, patients who experienced depression were 5.37 (95% CI [2.19–13.15]) times more likely to have cognitive symptoms than those who did not experience it, with 6.72 (95% CI [2.81–16.09]) greater chance of having concentration problems; 3.76 (95% CI [1.60–8.87]) greater chance of reporting memory problems, 2.81 (95% CI [1.18–6.70]) greater chance of reporting word finding difficulties, and 4.12 (95% CI [1.66–10.22]) greater chance of having difficulty thinking clearly. These results agree with a large longitudinal analysis of 1,733 consecutive patients with laboratory-confirmed COVID-19, in which 23% of patients reported concomitant symptoms of anxiety/depression 6 months after acute SARS-CoV-2 infection (44).

A systematic review that analyzed eight studies found considerable rates of depressive symptoms and clinically significant depression in post-COVID-19 syndrome. The frequency of depressive symptoms more than 12 weeks after a SARS-CoV-2 infection ranged between 11 and 28%. Two separate studies investigated the association between depression and neurocognitive functioning in post-COVID-19 syndrome and found that patients with depression tended to perform worse on neurocognitive tests compared to those without depression. Baseline markers of systemic inflammation and its change over time have been shown to predict depressive symptoms at three months of post-discharge follow-up. However, it remains to be seen whether the high frequency of depression among individuals with post-COVID-19 syndrome is a long-term consequence of the viral infection or a result of social, economic, and spatial factors (45). In the subanalysis excluding patients with depressive symptoms, we have obtained very similar results to the analysis using the full sample size. In other words, despite the overlap, post-COVID cognitive symptoms are not always linked to depression.

No association was found between the occurrence of persistent cognitive symptoms and the patient’s vaccination status, although the impact of vaccination on post-COVID-19 syndrome differs across studies. An exploratory, observational single-center cohort study of patients hospitalized for COVID-19 demonstrated that vaccinated patients have a lower risk of developing impaired concentration (OR 0.49 [95% CI 0.24–0.98]) (37). Similarly, other studies also evidenced that vaccination reduces the risk of post-COVID outcomes (46) as reported in a meta-analysis, in which people who received two doses of vaccine were significantly less likely to develop this condition than unvaccinated people (47). With the widespread dissemination of COVID-19 vaccination, however, the evolving landscape necessitates further in-depth study. As vaccination reduces COVID-19 severity (48), there might be a positive impact in reducing persistent cognitive symptoms.

When evaluating cognitive symptoms, it is also important to understand their impact on patients’ daily activities, more specifically in their professional lives, but evidence of this impact is still scarce. In the present study, patients with cognitive symptoms reported a higher frequency of time away from work longer than the usual period of isolation, fourteen, ten or seven days since the onset of symptoms, depending on the protocol used and the moment of the pandemic (7.0 vs. 1.7%), and restrictions on returning to work (4.0 vs. 1.4%, *p* < 0.001 for both). A previous study focused on evaluating the quality of life at work in 300 patients before COVID-19 up to over 12 weeks post-acute infection. Only 44.67% of patients presented a normal quality of life at work after 12 weeks of COVID-19 diagnosis, and the authors observed that memory and focus impairment after 12 weeks of COVID-19 diagnosis was a predictor of poor quality of life at work (49).

The main limitation of this study is its reliance on the report of cognitive symptoms and the lack of formal neuropsychological assessment. Self-report measures can be influenced by different factors, including mood status. For instance, depression is associated with cognitive complaints not necessarily confirmed by objective assessment (50). Nevertheless, we have confirmed a high frequency, even in patients without depressive symptoms. Other validated self-report measures of cognition could have been also used, for example, the Subjective Cognitive Decline Questionnaire (SCD-Q) (54). However, we opted for not including it to avoid the research questionnaire being extremely long, which could compromise data quality and response rate (51). The “temporal report” and recall biases may also be seen as potential limitations of the study. Additionally, other authors have already highlighted differences in the definitions of cognitive dysfunction, “brain fog,” memory issues and attention disorder (8). Conversely, the homogeneity of the studied population constitutes a strength of this study.

Future studies must map specific cognitive deficits (e.g., attention, memory, executive function) using quantitative neuropsychological tests. Additionally, future studies are needed to better understand the underlying mechanisms of post-COVID cognitive symptoms, so effective therapeutic approaches can be developed in order to improve quality of life and to mitigate disease burden.

## Conclusion

In this retrospective cohort of patients with mostly mild COVID-19, we have demonstrated that cognitive symptoms were common components of post-COVID-19 syndrome, present in 23.7% of patients at 12 weeks after acute COVID-19. Female sex, fatigue, depression and the need to seek in-person care during acute COVID-19 were independently associated with a higher risk for this condition.

## Data availability statement

The original contributions presented in the study are included in the article/Supplementary material, further inquiries can be directed to the corresponding author.

## Ethics statement

This study was approved by the Brazilian National Commission for Research Ethics (Comissão Nacional de Ética em Pesquisa on number CAAE 30350820.5.1001.0008), and it was conducted in accordance with the Declaration of Helsinki. All patients gave informed consent to participate in the study.

## Author contributions

LB: Writing – original draft, Writing – review & editing, Conceptualization, Data curation, Project administration. TC: Writing – original draft, Writing – review & editing, Data curation. BF: Writing – original draft, Data curation, Writing – review & editing. TP: Writing – original draft, Writing – review & editing, Data curation. DP: Writing – original draft, Writing – review & editing, Data curation. TF: Writing – original draft, Writing – review & editing, Data curation. LK: Writing – original draft, Writing – review & editing, Data curation. CO: Writing – original draft, Writing – review & editing, Conceptualization. AT: Writing – original draft, Writing – review & editing, Conceptualization. MM: Writing – original draft, Writing – review & editing, Conceptualization, Data curation, Project administration.

## Glossary

**Table:** 

CI	Confidence Interval
CNS	Central Nervous System
COVID-19	Coronavirus Disease 2019
GAD-7	Generalized Anxiety Disorder–7
HIV	Human Immunodeficiency Virus
IQR	Interquartile Range
ME/CFS	Myalgic Encephalomyelitis/Chronic Fatigue Syndrome
NICE	National Institute for Health and Care Excellence
OR	Odds Ratio
PCL-5	Posttraumatic Stress Disorder Checklist for DSM-5
PHQ-2	Patient Health Questionnaire-2
PHQ-9	Patient Health Questionnaire-9
RT-PCR	Reverse Transcription-Polymerase Chain Reaction
SARS-CoV-2	Severe Acute Respiratory Syndrome Coronavirus 2
SCD-Q	Subjective Cognitive Decline Questionnaire
TNMG	Telehealth Network of Minas Gerais
UFMG	Universidade Federal de Minas Gerais
