# Supplementary material for: Post-COVID-19 cognitive symptoms in patients assisted by a teleassistance service: a retrospective cohort study
Source: Front Public Health. 2024 Apr 16;12:1282067. doi: 10.3389/fpubh.2024.1282067 (PMC11060150; doi:10.3389/fpubh.2024.1282067)
Supplement: Supplementary file 1 [file Data_Sheet_1.docx]

**Supplementary file 1** Post-COVID-19 symptoms questionnaire.

**POST-COVID-19 SYMPTOMS QUESTIONNAIRE**

1. Researcher’s email:

2. Researcher’s name:

“x”

“y”

“z”

Other

3. If other researchers, who?

Before we start with the questions, I will present to you some important information regarding our study.

*Mandatory

Verbal consent form:

Sr/Ms. PATIENT’S NAME,

The present project aims to evaluate persistent symptoms after the acute phase of COVID-19 in patients from the Monitora-COVID/UFMG. This is crucial to help us define the need for the creation of specific attending flows. The results can bring benefits to patients that were diagnosed with COVID-19. The present study is conducted by the Telehealth Center of the University Hospital (UFMG) and was approved by the ethics committee. During the participation all of your information will be secured and no personal data will be shared. You may stop participating at any moment and request your collected data. Your participation will not bring you any burden. Your data will be secured in a University’s computer, without your name and may be anonymously used to aid in public policies or in scientific publications. We identified that you were attended to by the Monitora-COVID:

4. May we continue?

*Choose only one option.*

Yes, the patient will answer now.

*Skip to question 5*

Yes, the patient will answer later.

No.

The patient died.

Initial information

Firstly, I would like to confirm some of your data.

5. What is your CPF?

Check if the CPF is the same as the one in the sheets.

6. Which race do you identify?

*Choose only one option.*

White

Brown

Black

Asian

Indigenous

Would not like to declare

7. What is your scholar level?

*Choose only one option.*

Did not complete middle school

Completed middle school

High school

Graduate

Post-graduate

8. Are you a health professional, a student from a health-related area or are involved in the care of others (at home)?

*Choose only one option.*

Yes

No

9. How are you connected to UFMG?

*Choose only one option.*

Effective

EBSERH

Outsourced

Student

Resident

Intern

Other

10. If other, how?

Occupation

11. Where do you work?

If professional:

12. What is your work?

If student/intern/resident:

13. Which is your course?

Regarding the COVID-19 diagnosis

Now we will ask you some questions regarding the acute phase of COVID-19, when you were attended to by Monitora COVID.

14. Did you require any in-person consults for COVID-19 treatment after being attended by TeleCOVID?

*Choose only one option.*

Yes

No

15. If yes, where?

*Choose only one option.*

Health center

Private physician's office

Emergency service

Public hospital

Private hospital

Other

16. If other, which?

For patients that received in-person consults:

17. During the teleconsult were you referred to in-person appointments?

*Choose only one option.*

Yes

No

18. Were you admitted to a hospital?

*Choose only one option.*

Yes

No

19. Were you admitted to an ICU?

*Choose only one option.*

Yes

No

20. Did you require mechanical ventilation?

*Choose only one option.*

Yes

No

21. Which is your birth sex?

*

*Choose only one option.*

Woman

*Skip to question 22*

Man

*Skip to section 6 (Post-COVID-19 symptoms)*

Would not like to declare

*Skip to question 22*

Pregnancy

22. During the acute phase of COVID-19 (up to a month after the beginning of symptoms), were you at any stage of pregnancy or puerperium (up to two weeks postpartum, including fetal loss or abortion?

*Choose only one option.*

Yes

No

Post-COVID-19 symptoms

Mr/Ms PATIENTS NAME,

We will now ask questions regarding symptoms that you may have presented during COVID-19 and you need to answer if you presented or did not present the symptom and their duration.

Beginning of the symptoms

23. Did the first symptom start over six months ago?

*Choose only one option.*

Yes

No

Respiratory manifestations

24. Did you cough?

*Choose only one option.*

Yes, up to a month

Yes, up to three months

Yes, up to six months

Yes, it persisted after six months

No

25. Did you have rhinorrhea or a nose itch??

*Choose only one option.*

Yes, up to a month

Yes, up to three months

Yes, up to six months

Yes, it persisted after six months

No

ATTENTION!

Verify if the patient has a history of rhinitis.

26. Did you present thoracic or chest pain?

*Choose only one option.*

Yes, up to a month

Yes, up to three months

Yes, up to six months

Yes, it persisted after six months

No

27. Did you feel shortness of breath?

*Choose only one option.*

Yes, up to a month

*Skip to question 31*

Yes, up to three months

*Skip to question 28*

Yes, up to six months

*Yes, skip to question 28*

Yes, it persisted after six months

*Skip to question 28*

No

*Skip to question 31*

Shortness of breath degree

We will evaluate the degree (intensity) of your shortness of breath. Considering the second month after COVID-19 diagnosis (after the first month- acute phase), please answer the following:

28. When did you present shortness of breath?

*Choose only one option.*

In extra-ordinary activities, such as running, carrying heavy weight in pain field or light weights climbing up stairs

With more intense activities, such as climbing up inclined hills, two or more levels or carrying a package of groceries in a plain field

With moderate activities, such as showering, walking a block at a regular pace

While resting or when dressing or walking a few slow steps

29. Did you have to measure your oxygen saturation at home?

*Choose only one option.*

Yes

No

30. If so, were the values persistently lower than 95%?

*Choose only one option.*

Yes

No

Musculoskeletal alterations

31. Did you present any alterations in smell?

*Choose only one option.*

Yes, up to a month

Yes, up to three months

Yes, up to six months

Yes, it persisted after six months

No

32. Did you present any alterations in taste?

*Choose only one option.*

Yes, up to a month

Yes, up to three months

Yes, up to six months

Yes, it persisted after six months

No

33. Did you present joint pain?

*Choose only one option.*

Yes, up to a month

Yes, up to three months

Yes, up to six months

Yes, it persisted after six months

No

34. Did you present muscular pain?

*Choose only one option.*

Yes, up to a month

Yes, up to three months

Yes, up to six months

Yes, it persisted after six months

No

35. Did you present numbness, tingling or alterations in sensibility?

*Choose only one option.*

Yes, up to a month

Yes, up to three months

Yes, up to six months

Yes, it persisted after six months

No

36. Did you have a stroke?

*Choose only one option.*

Yes, up to a month

Yes, up to three months

Yes, up to six months

Yes, it persisted after six months

No

Physical fatigue

We will ask questions regarding physical fatigue.

37. Did you get tired easily?

*Choose only one option.*

Yes, up to a month

Yes, up to three months

Yes, up to six months

Yes, it persisted after six months

No

38. Did you need to rest more often?

*Choose only one option.*

Yes, up to a month

Yes, up to three months

Yes, up to six months

Yes, it persisted after six months

No

39. Were you sleepy?

*Choose only one option.*

Yes, up to a month

Yes, up to three months

Yes, up to six months

Yes, it persisted after six months

No

40. Did you have any difficulties in beginning any activities?

*Choose only one option.*

Yes, up to a month

Yes, up to three months

Yes, up to six months

Yes, it persisted after six months

No

41. Did you feel low?

*Choose only one option.*

Yes, up to a month

Yes, up to three months

Yes, up to six months

Yes, it persisted after six months

No

42. Did you feel muscular weaknesses?

*Choose only one option.*

Yes, up to a month

Yes, up to three months

Yes, up to six months

Yes, it persisted after six months

No

43. Did you feel weakened?

*Choose only one option.*

Yes, up to a month

Yes, up to three months

Yes, up to six months

Yes, it persisted after six months

No

Mental fatigue

In regards to mental fatigue.

44. Did you present alterations in concentration?

*Choose only one option.*

Yes, up to a month

Yes, up to three months

Yes, up to six months

Yes, it persisted after six months

No

45. Did you present difficulties in thinking clearly?

*Choose only one option.*

Yes, up to a month

Yes, up to three months

Yes, up to six months

Yes, it persisted after six months

No

46. Did you present word finding difficulties?

*Choose only one option.*

Yes, up to a month

Yes, up to three months

Yes, up to six months

Yes, it persisted after six months

No

47. Did you present memory loss?

*Choose only one option.*

Yes, up to a month

Yes, up to three months

Yes, up to six months

Yes, it persisted after six months

No

Neuro-cognitive manifestations

We will now ask questions concerning the nervous system.

Observation:

In regards to the duration of symptoms:

Always consider the duration from the first day of symptoms.

48. Did you have any bad memories or negative emotions related to the episode of COVID-19?

*Choose only one option.*

Yes, up to a month

Yes, up to three months

Yes, up to six months

Yes, it persisted after six months

No

49. Did you present little interest or pleasure in doing things?

*Choose only one option.*

Yes, up to a month

Yes, up to three months

Yes, up to six months

Yes, it persisted after six months

No

50. Did you feel isolated from other people?

*Choose only one option.*

Yes, up to a month

Yes, up to three months

Yes, up to six months

Yes, it persisted after six months

No

51. Did you have any trouble sleeping, or were you sleeping without interruptions or sleeping more than what you are used to?

*Choose only one option.*

Yes, up to a month

Yes, up to three months

Yes, up to six months

Yes, it persisted after six months

No

52. Did you feel nervous, anxious or irritated?

*Choose only one option.*

Yes, up to a month

Yes, up to three months

Yes, up to six months

Yes, it persisted after six months

No

53. Were you unable to stop worrying or to control your worries?

*Choose only one option.*

Yes, up to a month

Yes, up to three months

Yes, up to six months

Yes, it persisted after six months

No

54. Did you feel low, discouraged or with a lack of hope?

*Choose only one option.*

Yes, up to a month

Yes, up to three months

Yes, up to six months

Yes, it persisted after six months

No

55. Were you unable to do your daily life activities (organizing, planning, problem-solving, decision-making or data processing)?

*Choose only one option.*

Yes, up to a month

Yes, up to three months

Yes, up to six months

Yes, it persisted after six months

No

56. If you presented any of the previous symptoms: did they make your daily activities more difficult?

*Choose only one option.*

Yes

No

Not applicable

57. Did you begin psychological treatment with or without prescribed medications for psychological issues after COVID-19?

*Choose only one option.*

Yes

No

Other Manifestations

58. Did you present thrombosis or pulmonary embolism?

*Choose only one option.*

Yes, up to a month

Yes, up to three months

Yes, up to six months

Yes, it persisted after six months

No

59. Did you present hair loss?

*Choose only one option.*

Yes, up to a month

Yes, up to three months

Yes, up to six months

Yes, it persisted after six months

No

Comorbidities

We will ask about chronic conditions that you may have.

60. Do you have obesity?

*Choose only one option.*

Yes

No

61. Did you have a stroke before COVID-19?

*Choose only one option.*

Yes

No

62. Do you have any chronic diseases?

*Choose only one option.*

Yes

*Skip to question 63*

No

*Skip to question 69*

Comorbidities

63. Do You have any chronic diseases?

*Choose all that apply.*

Cardiac arrhythmia (atrial fibrillation, flutter)

Asthma/Bronchitis

Cirrhosis

Coronary artery disease

Chronic obstructive pulmonary disease

Heart failure

Renal failure

Hypertension

Active tuberculosis

Previously treated tuberculosis

Cancer

Other

64. If others, which ones?

65. Do you have diabetes?

*Choose only one option.*

Yes

*Skip to question 66*

No

*Skip to question 69*

Diabetes mellitus

66. If you do have diabetes, did you require insulin before COVID-19?

*Choose only one option.*

Yes

No

If you used insulin prior to COVID-19:

67. Did you need higher doses of insulin after COVID-19?

*Choose only one option.*

Yes

No

If you did not use insulin prior to COVID-19:

68. Did you require insulin after COVID-19?

*Choose only one option.*

Yes

No

Medications

Now, regarding the use of medications.

69. Do you take any medications?

*Choose only one option.*

Yes

No

70. If so, which ones?

Life habits

We will now ask about your like habits.

71. Do you or did you ever smoke?

*Choose only one option.*

Yes

No

72. Do you practice any physical activities?

*Choose only one option.*

Yes

No

Work impact

We will now ask about COVID-19’s impact on your work.

73. Were you off from work after the usual period of isolation?

*Choose only one option.*

Yes

No

74. If yes, for how long (in days)?

75. If yes, why?

76. Were you relocated to remote work after the isolation period?

*

*Choose only one option.*

Yes

No

Did not have the option

77. If yes, why?

78. If yes, for how long (in days)?

79. Houve necessidade de restrição(ões) para a realização das atividades habituais de trabalho após o retorno do período de isolamento?

*Choose only one option.*

Yes

No

Did not have the option

80. If yes, which ones?

81. If yes, for how long (in days)?

Functionality post-COVID-19

We will evaluate how much you are currently affected in your daily life by COVID-19.

82. Choose which of the following affirmations best applies to you.

*Choose only one option.*

There are no limitations in my daily life and no symptoms related to the infection

There are insignificant limitations in my daily life, since I am able to do all usual activities, although I present symptoms

I have limitations in my daily life and occasionally need to avoid or reduce usual activities, or have the need to elongate activities due to the symptoms. However, I am capable of doing all daily activities without the need for assistance.

I have limitations to my daily life and am not able to do all usual activities due to symptoms. However, I am able to take care of myself and do not require assistance.

I present limitations in my daily life that require assistance due to the presence of symptoms.

Do you have any observations?

Thank you for your participation!

Your contribution will be very useful for studies concerning post-COVID-19 symptoms.

83. General or specific observations on his interview:
