# Supplementary material for: Post-COVID-19 cognitive symptoms in patients assisted by a teleassistance service: a retrospective cohort study
Source: Front Public Health. 2024 Apr 16;12:1282067. doi: 10.3389/fpubh.2024.1282067 (PMC11060150; doi:10.3389/fpubh.2024.1282067)
Supplement: Supplementary file 2 [file Data_Sheet_2.docx]

**Supplementary file 2** Data collection protocol.

**DATA COLLECTION PROTOCOL**

**GUIDANCE MANUAL FOR APPLICATION OF QUESTIONNAIRE BY PHONE**

RESEARCH TEAM

Supervisor: Prof. Milena Soriano Marcolino – [milenamarc@gmail.com](mailto:milenamarc@gmail.com)

Co-supervisor: Prof. Clara Rodrigues Alves de Oliveira – [claralves@gmail.com](mailto:claralves@gmail.com)

Co-supervisor: Prof. Thais Rotsen Correa – [thaisrotsencorrea@gmail.com](mailto:thaisrotsencorrea@gmail.com)

Senior researcher: Lívia Paula Freire Bonfim – [lpfbonfim@yahoo.com.br](mailto:lpfbonfim@yahoo.com.br)

Collaborator: Luciane Kopittke – [lucianekopittke@gmail.com](mailto:lucianekopittke@gmail.com)

Undergraduate students:

Bruno Cabaleiro Cortizo Freire – [brunocabaleirocf@gmail.com](mailto:brunocabaleirocf@gmail.com)

Daniella Nunes Pereira – [dani.nunesp2511@gmail.com](mailto:dani.nunesp2511@gmail.com)

Lucca Fagundes Ramos de Oliveira – [lucca.oliveira@aluno.ufop.edu.br](mailto:lucca.oliveira@aluno.ufop.edu.br)

Thais Marques Pedroso – [thais.marques.pedroso@gmail.com](mailto:thais.marques.pedroso@gmail.com)

Thalita Baptisteli Fernandes – [tatabf99@gmail.com](mailto:tatabf99@gmail.com)

**GENERAL GUIDELINES FOR RESEARCHERS**

The **GUIDANCE MANUAL** serves to clarify your doubts and **MUST ALWAYS BE WITH YOU**. Consult the manual **WHENEVER IT BECOMES NECESSARY**. Reread the manual **PERIODICALLY**. Avoid relying excessively on your memory.

Here we present the general guidelines on how to approach the participant. These instructions are **VERY IMPORTANT** and guide the conduct of the researcher throughout the entire job.

- **Keep a good relationship with the research team.** Remember everyone is **essential to the success of the project.**
- Communicate with team members and share questions and suggestions via instant text messages app (*Whatsapp®*), phone or email.
- Be proactive. Always be kind and polite, as the participant has no obligation to enter the survey. **Watch out for language and vocabulary**. The impression aroused in the participant is **very important** for the accomplishment of the work.
- Always treat participants with respect. When initiating your contact, ask him/her: “How do you prefer to be treated?” Address properly and always call him/her by name (eg. Ms Joana) throughout the interview. If he/she doesn't make any choice, treat him/her formally.
- Right from the start, it is important to establish a cordial dialogue with the participant, treating him with respect and attention. **Never** show haste or impatience following hesitation or delay in answering a question. Wait for the responses to finish. Even if there is a hesitation period, do not interrupt the participant.
- Always identify yourself. Remind the participant that the researchers are available for the clarification of doubts and that you will also be available for any doubts later.
- Do not eat or drink during the interview.
- **Reserve your place and time of work in advance. Prepare the space with conditions and materials you may need (desk, computer, paper, pen, spreadsheets, telephone, silence). Strive to be punctual and fulfill the agreed workload.**
- During the telephone call, preferably use a headset, keep your hands free, keep the computer on, with the pre-filled questionnaire on the screen, and keep the worksheets and pencil/pen within reach. Study the Monitora service data about the participant before attempting contact.
  1. **GUIDELINES FOR PATIENT RECRUITMENT**

*** BEFORE STARTING RECRUITMENT:**

- Before attempting to call, every researcher will receive a list of patients for whom will be responsible. This list will contain all the information necessary to contact the patient (name, cell phone, main TeleCOVID-MG-MG service data).
- All patients who fulfill the following requisites will be recruited:
  - Belong to the UFMG Community;
  - Were assisted by TeleCOVID-MG;
  - Presented a positive COVID-19 test (RT-PCR or antigen test).
- Write down on the spreadsheet the date of the patient’s first symptoms and the timeframes (1 month, 3 months, 6 months), according to the chronology of the patient’s disease, to guide the questions related to the disease periods. For example, if the first day of symptoms was 01/01/2020, fill in the spreadsheet:
  - First day: 01/01/20201
  - Up to 1 month: until 31/01/2020
  - Up to 3 months: until 31/03/2020
  - Up to 6 months: until 30/06/2020
  - More than 6 months: above 30/06/2020
- Recruitment will take place by telephone (one or more contacts, if needed), according to the instructions below:

* **INTRODUCTORY CALL (1^st^ CONTACT)**

- Hello, good morning/afternoon/evening (patient’s name). My name is [...], I am an undergraduate medical student and I am a part of a project which aims to evaluate symptoms that persist after the acute phase of COVID-19 in patients assisted by TeleCOVID-MG. This project will be important to define the need to create specific flows for care. May I talk to you about this?

*** If the answer is no:**

- Finish the contact and appreciate the attention. Let him know that if he eventually changes his mind, he will be able to participate. Show yourself available for a new conversation.

*** If the answer is yes:**

- Initiate the questionnaire.

*** IF YOU CANNOT REACH THE PATIENT:**

- Try calling again another time. Always respect the business hours, between 08:00h and 18:00h.
  - If you called once in the morning shift and again in the afternoon shift, and the contact was also unsuccessful, send a protocol instant text message via *Whatsapp®.*

If you cannot reach the patient after both telephone calls and text messages, finish the contact.

1. **Protocol for instant text messages via Whatsapp®.**

Dear Mr/Ms (PATIENT’S NAME),

I hope you are fine and healthy.

My name is (RESEARCHER’S NAME), I am an undergraduate Medical student and I am participating in a COVID-19 study.

This study is being conducted by the telehealth centre from the Hospital das Clínicas da UFMG.

May you inform us of the best time to contact you via a telephone call, please?

Thank you!

1. **Patient recruitment (Flowchart):**

*
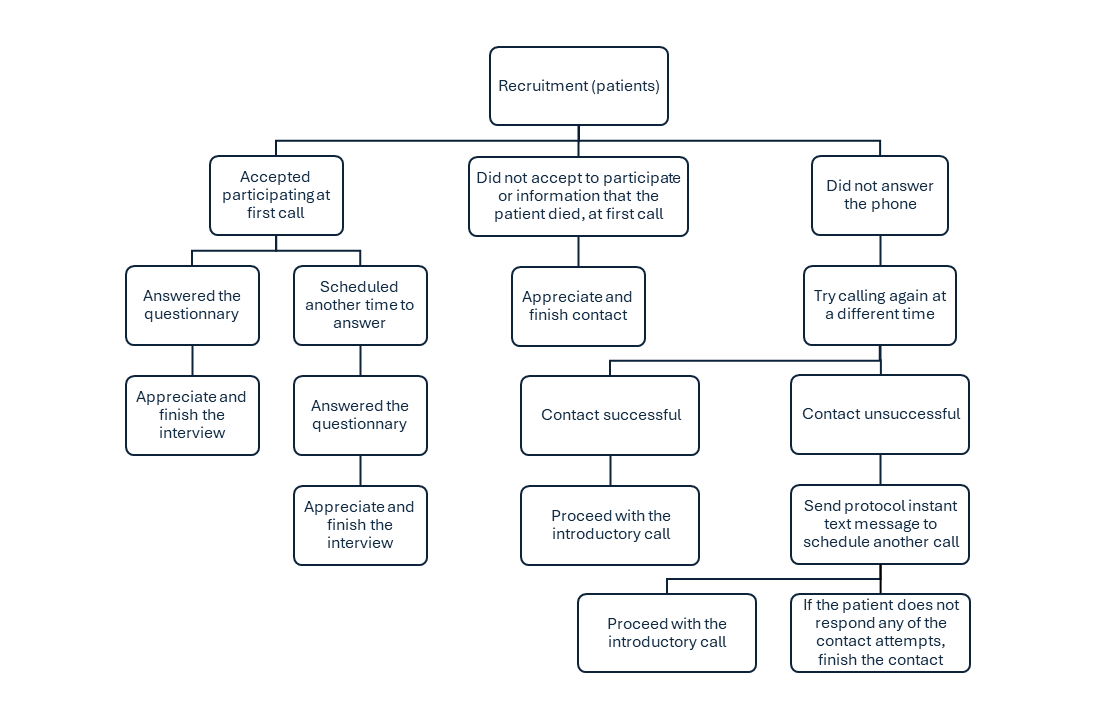
*

- 1. **GUIDELINES FOR OBTAINING THE INFORMED CONSENT**

Mr./Ms. (patient’s name),

Before initiating the questionnaire, I will explain to you some important information about our study:

This project aims to assess symptoms that persist after the acute phase of COVID-19 in people attended by TeleCOVID-MG and will be important to define the need to create specific flows for care. The results of this study may result in benefits for patients who have had COVID-19.

This work is being carried out by the Telehealth Center of the University Hospital (UFMG) and was approved by the Research Ethics Committee.

During participation, all information about you will be confidential and no personal data will be disclosed. You may withdraw your participation at any time and request your information from researchers whenever you want. Your participation will not incur any cost to you. Your data will be stored on a UFMG computer without your name and can be used to assist public policies or in scientific publications, always anonymously.

We identified that you were attended by TeleCOVID-MG.

Do you agree to participate? May we proceed?

*** If the answer is no:**

- Finish the contact and appreciate the attention. Let him know that if he eventually changes his mind, he will be able to participate. Show yourself available for a new conversation.

*** If the answer is yes:**

- Initiate the questionnaire.
  1. **GENERAL GUIDELINES FOR THE APPLICATION OF THE QUESTIONNAIRE:**

● When calling the patient, have the questionnaire open on the computer.

● Before calling, study the patient care data provided by the TeleCOVID-MG record, contained in the general spreadsheet and complete the columns of the service worksheet.

● Ask the questions in the **order** they appear on the test.

● Ask the questions **exactly** as they are phrased on the test in a **neutral tone**: even small

changes can change the answer obtained.

● During the interview, sometimes refer to the patient's name. It is a

strategy to get attention and maintain the patient's interest. For example: “Mr John, now

let's talk about…” and not simply “Now let's talk about…”.

● Try to make the dialogue dynamic; show interest in what is being reported.

● It is essential that you have a **deep** knowledge of the content of the test you are going to apply as well as your instruction manual, be fully familiar with the terms used in the interview and ensure there is no doubt or hesitation on your part when asking questions. Only the patient has the right to hesitate.

● Be clear in the wording of the questions, according to the test’s model. Strictly follow the guidelines. If the patient doesn't understand, **repeat the question**. Only if it persists, you should explain what you want to know with that question.

● All questions must be read to the patient, even if the information has already been spontaneously given by him/her. In this case, make it clear that you know this answer has already been mentioned, but that it is still necessary to ask the question, for example, saying “just to confirm”.

**● If the participant answers “I don't know” to a question related to time, say the next: So let's recap. Your symptoms started on the day (have this information by hand). Until which month did the cough last more or less? Use this to calculate the time. People won't remember, retrospective study is complicated, and we need to help.**

● **Never** influence or suggest responses.

● **Never show disapproval, approval, or surprise at responses.** Remember that the purpose of the interview is to obtain information and not to judge, impart teachings or influence people's conduct. The researcher's posture should always be **neutral** concerning the answers. Leave the surprise and your disquiet about the answers to discuss with the supervisor.

● When the patient asks something that the researcher does not know how to answer, the question should be written down and taken as quickly as possible to the senior researcher, to then schedule another call with the patient.

● Always clarify doubts and explain what the patient does not understand.

● Try to maintain an open dialogue with the senior researcher, immediately reporting any problem, difficulty or doubt that arises during the training and interviews. **Your doubts** are important to improve the group**'**s work. Always clarify your doubts before moving on to the next call.

● Be aware of patients with special needs. If patients with special needs are interested in participating in the study, it must be ensured that they can participate and, later, you must consult the supervisor to assess possible limitations of his/her participation. For example:

- - Only raise or lower the tone of voice if the patient requests it;
  - Be careful with people who have speech difficulties (eg stuttering);
  - Try to make the patient comfortable, and speak slowly, in a didactic and contextualized way.

● Be kind and appreciate participating in the survey.

● Always return to the questionnaire and manual in case of forgetfulness or error.

● Take good care of worksheets and the quiz link. They will be used during all interviews.

● All information recorded in the open field must be filled in clearly and objectively.

● **Always keep your GUIDANCE MANUAL at hand** and do not hesitate to consult it,

if necessary, during the interview.

● At the end of each call, complete the worksheet.

- 1. **SPECIFIC GUIDELINES FOR THE APPLICATION OF THE QUESTIONNAIRE:**

● The purpose of this questionnaire is to evaluate the occurrence/persistence of post-acute (one month after the onset) COVID-19 symptoms, in patients from the community of the UFMG, attended by TeleCOVID-MG and who tested positive for SARS-Cov-2 (RT-PCR or antigen test).

● Before starting the test application, open the TeleCOVID-MG data sheet and fill in the worksheet. Proceed to the questions and record the answers. Study the information related to the service given by TeleCOVID-MG.

● Before starting, tell the patient:

- (Patient's name), I would like to reinforce that in each of the questions, you must choose only one of the answer alternatives, which according to your opinion is the one that is the closest to your reality. May we proceed?

● During the application of the questionnaire, it is important to read each question, always with a pause between the statement of the question and the alternative answers, to facilitate the patient's understanding.

● If the patient has doubts about a term found in any question, those doubts must be clarified until the patient completely comprehends and the question can be answered with conviction.

Exemple: If the research participant does not understand the question “Did you have difficulty thinking clearly?”, the interviewer should explain the symptom: "having difficulty maintaining a line of thought, or having 'blank' spaces when thinking; comparing how clear your current thinking is compared with its previous functioning". Likewise, if the interviewee does not understand the question “Did you have word finding difficulties?”, the researcher had to explain: "not remembering the word he/she wanted to say”.

● Always resolve all doubts (with the senior researcher) before moving on to the next interview.

● Before starting to fill in the form:

1. Read and follow the guidance manual;

2. Open the spreadsheet (general) and study the information of the patient to be interviewed;

3. Fill in the columns of the service worksheet (patients list). Complete with the date of onset of symptoms for the patient to be interviewed, as well as 1-month, 3-month, and 6-month timeframes (from the onset of symptoms) to ease the conduction of questions related to the duration of symptoms;

4. Confirm the patient's CPF during the interview. The CPF must be correct and identic to the spreadsheet, containing only numbers, without other characters.

6. At the end of the interview, complete the attendance sheet.

FORMULARY:

**Post-COVID-19 symptoms**

Mr./Ms. (patient’s name),

Now let’s talk about the symptoms you presented during the episode of COVID-19. I will ask about some symptoms. You shall answer if you had or not the symptom.

**ATTENTION!**

For questions about the occurrence and duration of symptoms:

- First, ask if there was:

- If not, move on to the next symptom. You don't need to read all the symptom duration options.

- If yes, read the symptom duration options and mark the most appropriate. In that case, be prepared with the date of the first symptom, and the timeframes, to help the participant to answer correctly.

**Respiratory manifestations**

Let's start by talking about respiratory symptoms!

Did you have a cough?

If yes, read the options.

If not, move on to the next symptom.

Do this for all questions about symptoms and their duration.

...

Have you had coryza (fluid and clear secretion) and/or an itchy nose?

Observation:

When asking about the occurrence of coryza (fluid and clear secretion) and/or itchy nose, if the answer is yes, ask if the patient has rhinitis. If not, keep the previous answer. If the patient reports that he has rhinitis, ask if the symptoms were similar to the usual or if they were different and/or more intense. If they were similar to the usual ones (out of the COVID-19 disease), tick no. If they are different and/or more intense, tick yes.

**Medications**

In this section, if the applicator notices regular use of medications, and the patient has responded "no" for the presence of chronic disease, recheck the presence of comorbidities, return to the question "Do you have any chronic illness?" and mark "yes".

Often the patient refers that he does not have "high blood pressure", since the pressure is controlled due to the use of antihypertensives.

In this case, tick "yes" for chronic disease and "yes" for systemic arterial hypertension.

**Life habits**

Observation:

– Examples of moderate-intensity physical activities: walking at > 5 km/h, cycling at less than 16 km/h, playing doubles tennis and hall dancing.

– Examples of vigorous-intensity physical activities: running, walking on slopes, cycling at more than 16 km/h, and aerobic dancing.

Any questions, contact the senior researcher, at any time during the process.

**Have a nice work!!!**
