# Supplementary material for: Post-COVID-19 cognitive symptoms in patients assisted by a teleassistance service: a retrospective cohort study
Source: Front Public Health. 2024 Apr 16;12:1282067. doi: 10.3389/fpubh.2024.1282067 (PMC11060150; doi:10.3389/fpubh.2024.1282067)
Supplement: Supplementary file 3 [file Table_1.docx]

**Supplementary table 1** Categorization of variables used in statistical analysis.

| **Variable category** | **Variables** |
| --- | --- |
| Possible predictor of cognitive symptoms | Age, sex, race, education, healthcare professional, vaccination status, COVID wave, comorbidities, smoking, sedentary lifestyle, pregnancy, needed to seek in-person care, hospital admission and depression symptoms. |
| Likely consequence of cognitive symptoms or other post-COVID-19 manifestations | Loss of ability to carry out daily tasks, time away from work longer than the usual period of isolation, restrictions on returning to work and post-covid functional status. |
