# Supplementary material for: Post-COVID-19 cognitive symptoms in patients assisted by a teleassistance service: a retrospective cohort study
Source: Front Public Health. 2024 Apr 16;12:1282067. doi: 10.3389/fpubh.2024.1282067 (PMC11060150; doi:10.3389/fpubh.2024.1282067)
Supplement: Supplementary file 4 [file Table_2.docx]

**Supplementary table 2** Clinical and social characteristics from the study group, comparing patients with and without cognitive symptoms.

| **Characteristics** | **Total sample**  **(n=630)** | | **Cognitive symptoms**  **(n=149)** | | **No cognitive symptoms**  **(n=481)** | | ***p-value*** |
| --- | --- | --- | --- | --- | --- | --- | --- |
| *Age group, years* |  |  |  |  |  |  | 0.319 |
| 17-40 | 440 | (69.8) | 101 | (67.8) | 339 | (70.5) |  |
| 41-60 | 176 | (27.9) | 46 | (30.9) | 130 | (27.0) |  |
| >60 | 12 | (1.9) | 1 | (0.7) | 11 | (2.3) |  |
| Missing data | 2 | (0,3) | NA | | NA | |  |
| *Comorbidities*^a^ |  |  |  |  |  |  |  |
| Obesity | 67 | (10.6) | 17 | (11.4) | 50 | (10.4) | 0.726 |
| Hypertension | 44 | (7.0) | 9 | (6.0) | 35 | (7.3) | 0.605 |
| Hypothyroidism | 26 | (4.1) | 7 | (4.7) | 19 | (4.0) | 0.688 |
| Diabetes mellitus | 18 | (2.9) | 4 | (2.7) | 14 | (2.9) | 0.885 |
| Connective Tissue Disease | 14 | (2.2) | 3 | (2.0) | 11 | (2.3) | 0.843 |
| Chronic cardiac disease | 4 | (0.6) | 0 | 0 | 4 | (0.8) | NA |
| HIV | 4 | (0.6) | 1 | (0.7) | 3 | (0.6) | NA |
| Malignancy | 3 | (0.5) | 2 | (1.3) | 1 | (0.2) | NA |
| Chronic neurological conditions | 3 | (0.5) | 0 | 0 | 3 | (0.6) | NA |

Numbers: N (%). NA: not applicable (insufficient sample size to apply the chi-square test). ^a^No patients had chronic respiratory or renal disease, liver disease or dementia.

**Supplementary table 2** Clinical and social characteristics from the study group, comparing patients with and without cognitive symptoms (continuation).

| **Characteristics** | **Total sample**  **(n=630)** | **Cognitive symptoms**  **(n=149)** | **No cognitive symptoms**  **(n=481)** | ***p-value*** |
| --- | --- | --- | --- | --- |

| *Race* |  |  |  |  |  |  | 0.354 |
| --- | --- | --- | --- | --- | --- | --- | --- |
| White | 353 | (56.0) | 77 | (51.7) | 276 | (57.4) |  |
| Brown | 184 | (29.2) | 50 | (33.6) | 134 | (27.9) |  |
| Black | 77 | (12.2) | 20 | (13.4) | 57 | (11.9) |  |
| Undeclared | 10 | (1.6) | 2 | (1.3) | 8 | (1.7) |  |
| Yellow | 4 | (0.6) | 0 | 0 | 4 | (0.8) |  |
| Indigenous | 2 | (0.3) | 0 | 0 | 2 | (0.4) |  |
| *Education* |  |  |  |  |  |  | 0.344 |
| Graduation | 375 | (59.5) | 82 | (55.0) | 293 | (60.9) |  |
| Post-graduation | 223 | (35.4) | 57 | (38.3) | 166 | (34.5) |  |
| High school | 30 | (4.8) | 10 | (6.7) | 20 | (4.2) |  |
| Middle school | 2 | (0.3) | 0 | 0 | 2 | (0.4) |  |

Numbers: N (%). NA: Not applicable (insufficient sample size to apply the chi-square test).
