# Supplementary material for: Post-COVID-19 cognitive symptoms in patients assisted by a teleassistance service: a retrospective cohort study
Source: Front Public Health. 2024 Apr 16;12:1282067. doi: 10.3389/fpubh.2024.1282067 (PMC11060150; doi:10.3389/fpubh.2024.1282067)
Supplement: Supplementary file 5 [file Table_3.docx]

**Supplementary table 3** Incidence of cognitive symptoms in the study population according to COVID-19 pandemic waves.

|  | **Second wave (n=155)** | **Third wave**  **(n=474)** |
| --- | --- | --- |
| Cognitive symptoms at 12 weeks | 48 (31.0) | 101 (21.3) |
| No cognitive symptoms at 12 weeks | 107 (69.0) | 373 (78.7) |

Numbers: N (%).
