# Supplementary material for: Post-COVID-19 cognitive symptoms in patients assisted by a teleassistance service: a retrospective cohort study
Source: Front Public Health. 2024 Apr 16;12:1282067. doi: 10.3389/fpubh.2024.1282067 (PMC11060150; doi:10.3389/fpubh.2024.1282067)
Supplement: Supplementary file 6 [file Table_4.docx]

**Supplementary table 4** Predictors of cognitive symptoms according to the multivariate analysis

(n=605 – only patients without depressive symptoms).

| **Variable** | **At least one cognitive symptom** | **Concentration** | **Memory** | **Word finding difficulties** | **Thinking clearly** |
| --- | --- | --- | --- | --- | --- |
| Fatigue | 2.23  (1.14-4.37) | 3.06  (1.19-7.84) | 3.76  (1.47-9.62) | 2.30  (1.02-5.21) | 3.85  (1.17-12.68) |
| Women | 2.14  (1.33-3.43) | 2.38  (1.31-4.31) | 1.93  (1.13-3.30) | 2.11  (1.21-3.68) | NA |
| Needed to  seek in-person  care | 2.12  (1.21-3.72) | 1.96  (1.03-3.71) | 2.61  (1.44-4.72) | 2.40  (1.31-4.40) | NA |
| COVID wave  (second) | NA | NA | NA | NA | 2.36  (1.30-4.27) |

Numbers are presented as odds ratio (95% confidence interval); p<0.05. NA: not applicable; p>0.20 in univariate analysis.
